# Efficient Phase Unwrapping Architecture for Digital Holographic Microscopy

**DOI:** 10.3390/s111009160

**Published:** 2011-09-27

**Authors:** Wen-Jyi Hwang, Shih-Chang Cheng, Chau-Jern Cheng

**Affiliations:** 1 Department of Computer Science and Information Engineering, National Taiwan Normal University, Taipei, 117, Taiwan; E-Mail: jericho0703@gmail.com; 2 Institute of Electro-Optical Science and Technology, National Taiwan Normal University, Taipei, 117, Taiwan

**Keywords:** phase unwrapping, digital holographic microscopy, FPGA, reconfigurable computing, system on programmable chip

## Abstract

This paper presents a novel phase unwrapping architecture for accelerating the computational speed of digital holographic microscopy (DHM). A fast Fourier transform (FFT) based phase unwrapping algorithm providing a minimum squared error solution is adopted for hardware implementation because of its simplicity and robustness to noise. The proposed architecture is realized in a pipeline fashion to maximize throughput of the computation. Moreover, the number of hardware multipliers and dividers are minimized to reduce the hardware costs. The proposed architecture is used as a custom user logic in a system on programmable chip (SOPC) for physical performance measurement. Experimental results reveal that the proposed architecture is effective for expediting the computational speed while consuming low hardware resources for designing an embedded DHM system.

## 1. Introduction

Digital holographic microscopy (DHM) [1,2] is a highly effective means for non-invasively capturing the amplitude and phase data of an optically transparent or reflective specimen [3,4]. Digital holograms that contain information about the specimen can be recorded and obtained using a charge-coupled device (CCD) or a complementary metal oxide semiconductor (CMOS). However, the phase map derived from the reconstructed image of a digital hologram is non-linearly wrapped lying in the interval (−*π*,*π*]. To obtain the three-dimensional profile of a specimen, the wrapped phase map must be unwrapped to a continuous phase [5]. Such a phase unwrapping procedure is also important in other applications, including synthetic aperture radar interferometry (InSAR) [6] and magnetic resonance imaging (MRI) [7]. The phase unwrapping process may be performed offline in some of these applications, whose primary concern is the quality of the unwrapped phases. By contrast, for the DHM or electronic speckle pattern interferometry applications, in addition to accurate unwrapped phase reconstructions, fast phase unwrapping operation is desired [8–12] for attaining realtime video rendering with high frame rates.

A simple raster scan algorithm is able to perform realtime phase unwrapping. However, in the presence of noise, the raster-scan algorithm may lead to an accumulation of error that eventually results in large deviations near the end of the accumulation. Popular approaches to the robust phase unwrapping include least square techniques, where the unwrapped phase is obtained as the function whose discrete gradient has the least squares deviation from its available estimate. The Poisson equations are then derived for the optimization, which can be solved by the preconditioned conjugate gradient (PCG) [13,14] and Gauss–Seidel techniques [13,15]. Recent advances in phase unwrapping include the *ZπM* algorithm [16], which solves the problem as a sequence of binary optimization. Network programming techniques [16] and graph cut techniques [17] are then used for the optimization. Another efficient approach is to use branch cut technique for phase unwrapping, which can be implemented by hybrid genetic algorithms [18]. The common drawback of these techniques are that they are all iterative algorithms. The number of iterations may be high [19] and may vary [16,20] depending on the input wrapped phase map. For a realtime DHM, phase unwrapping algorithms with low and constant computational complexities are usually desired for providing fast video rendering with constant frame rate. Consequently, these robust phase unwrapping algorithms may make the design of a realtime DHM difficult. Moreover, for the embedded systems with limited computational capacities, the implementation of realtime robust phase unwrapping becomes a very challenging issue.

A number of implementations for fast phase unwrapping have been proposed [19–21]. The implementations presented in [20,21] are based on PCG [13,14] with field programmable gate array (FPGA) devices and graphic processing unit (GPU) [22] platforms, respectively. The implementation proposed in [19] is based on Gauss–Seidel techniques [13,15] with GPU platforms. Because both PCG and Gauss–Seidel techniques are iterative algorithms, only moderate acceleration is achieved. Moreover, some of these implementations are based on GPU, which may be difficult to be used for embedded devices due to high hardware cost and large power consumption.

The goal of this paper is to present a novel phase unwrapping hardware architecture for accelerating the computational speed of DHM. The algorithm [23] selected for the proposed architecture is also a least square technique for robust phase unwrapping. The algorithm is non-iterative, and therefore has constant computational complexity. As compared with its iterative least square counterparts [13–15], the selected algorithm is more computationally efficient [24,25]. In addition, it is based on fast Fourier transform (FFT), which can be efficiently implemented by hardware. Therefore, the algorithm is well-suited for the realtime DHM applications.

Based on the algorithm [23], the architecture is separated into four units: the pre-transform unit, the FFT unit, the post-transform unit, and the on-chip memory. The first three units are used for computation of the phase unwrapping algorithm. The on-chip memory is used for storing the source data and the intermediate results produced by these units so that the memory access time can be reduced. Novel pipeline architectures are proposed for the implementation of the pre-transform unit, the FFT unit, and the post-transform unit to maximize the throughput of the proposed architecture. Only a single multiplier and divider are used in the FFT unit and post-transform unit for lowering hardware resource utilization, respectively.

The proposed architecture has been implemented on FPGA devices so that it can operate in conjunction with a softcore CPU. Using the reconfigurable hardware, we are then able to construct a system on programmable chip (SOPC) system for the physical performance measurement for phase unwrapping in the embedded systems. As compared with its software counterpart running on Intel I-7 quad-core CPU, the proposed system has significantly lower computational time for phase unwrapping. In particular, when the image resolution is 513 × 513, the proposed system attains the speedup of 605 over its software counterpart. All these facts demonstrate the effectiveness of the proposed architecture.

## 2. The Phase Unwrapping Algorithm

This section briefly reviews the algorithm adopted for the hardware phase unwrapping implementation. Please refer to [23] for detailed discussion of the algorithm. Let *ζ*_*i*,*j*_ be the wrapped phase function of an unknown real-valued function *ϕ*_*i*,*j*_ for 0 ≤ *i* ≤ *N*, and 0 ≤ *j* ≤ *N*, where −*π < ζ*_*i*,*j*_ ≤ *π*, and *e*^*jζ*_*i*,*j*_^ = *e*^*jϕ*_*i*,*j*_^. Let *ψ*_*i*,*j*_ for 0 ≤ *i* ≤ 2*N*, and 0 ≤ *j* ≤ 2*N* be the periodic extension of *ζ*_*i*,*j*_ using the mirror reflection technique. That is,

(1) $$\psi_{i,j}=\{\begin{array}{ll} \zeta_{i,j} & \text{for }0\leq i\leq N,0\leq j\leq N,\\ \zeta_{2N-i,j} & \text{for }N<i\leq 2N,0\leq j\leq N,\\ \zeta_{i,2N-j} & \text{for }0\leq i\leq N,N<j\leq 2N,\\ \zeta_{2N-i,2N-j} & \text{for }N<i\leq 2N,N<j\leq 2N. \end{array}$$

Define

(2) $$\Delta_{i,j}^{x}=\psi_{i+1,j}-\psi_{i,j},\;\;\;\;\Delta_{i,j}^{y}=\psi_{i,j+1}-\psi_{i,j}$$

for all *i* and *j*. Note that these values must be computed as *wrapped* phase differences, where the values 2*π* or −2*π* will be added as necessary to ensure that $\Delta_{i,j}^{x}$ and $\Delta_{i,j}^{y}$ lie in the interval (−*π*, *π*].

Let *ϕ̄*_*i*,*j*_ be an estimation of *ϕ*_*i*,*j*_ based on *ζ*_*i*,*j*_. The goal of the phase unwrapping algorithm is to find *ϕ̄*_*i*,*j*_ minimizing

(3) $$\sum_{i,j}{\left({\bar{\phi }}_{i+1,j}-{\bar{\phi }}_{i,j}-\Delta_{i,j}^{x}\right)}^{2}+\sum_{i,j}{\left({\bar{\phi }}_{i,j+1}-{\bar{\phi }}_{i,j}-\Delta_{i,j}^{y}\right)}^{2}.$$

It can be shown that the optimal *ϕ̄*_*i*,*j*_ is the solution to

(4) $$({\bar{\phi }}_{i+1,j}-2{\bar{\phi }}_{i,j}+{\bar{\phi }}_{i-1,j})+({\bar{\phi }}_{i,j+1}-2{\bar{\phi }}_{i,j}+{\bar{\phi }}_{i,j-1})=\gamma_{i,j},$$

where

(5) $$\gamma_{i,j}=\Delta_{i,j}^{x}-\Delta_{i-1,j}^{x}+\Delta_{i,j}^{y}-\Delta_{i,j-1}^{y}.$$

Because both *ϕ̄*_*i*,*j*_ and *γ*_*i*,*j*_ are periodic, the Fourier transform can be used to solve Equation (4). Applying the 2N × 2N two-dimensional Fourier transforms to both sides of Equation (4) yields

(6) $$\Phi_{m,n}=\Gamma_{m,n}/(2\;\text{cos}(\pi m/M)+2\;\text{cos}(\pi n/N)-4),$$

where Φ_*m*,*n*_ and Γ_*m*,*n*_ are the Fourier transforms of *ϕ̄*_*i*,*j*_ and *γ*_*i*,*j*_, respectively. The function *ϕ̄*_*i*,*j*_ is obtained by the inverse Fourier transform to Equation (6). The estimated *ϕ*_*i*,*j*_ is then obtained by restricting the results to the grid defined by 0 ≤ *i* ≤ *N*, 0 ≤ *j* ≤ *N*.

Based on the discussions shown above, the phase unwrapping algorithm using the FFT is summarized as follows:

Step 0: Suppose *ζ*_*i*,*j*_, 0 ≤ *i* ≤ *N*, 0 ≤ *j* ≤ *N*, are given.
Step 1: Compute *γ*_*i*,*j*_, 0 ≤ *i* ≤ *N*, 0 ≤ *j* ≤ *N*, using Equation (5).
Step 2: Compute Γ_*m*,*n*_, 0 ≤ *i* < 2*N*, 0 ≤ *j* < 2*N*, using two-dimensional 2N × 2N fast Fourier transform (2D-FFT).
  The 2D-FFT operates as follows.
Step 2.1 For each row *i* of the array *γ*_*i*,*j*_, compute *λ*_*i*,*j*_, 0 ≤ *j* < 2*N*, using mirror reflection.
  That is, *λ*_*i*,*j*_ = *γ*_*i*,*j*_ for 0 ≤ *j* ≤ *N*, and *λ*_*i*,*j*_ = *γ*_*i*,2*N*−*j*_ for *N* ≤ *j <* 2*N*.
Step 2.2 Compute Λ_*i*,*n*_, 0 ≤ *n* < 2*N*, the FFT of *λ*_*i*,*j*_, 0 ≤ *j* < 2*N*.
Step 2.3 Replace *i*-th row of the array *γ*_*i*,*j*_ by Λ_*i*,*n*_ with the restriction that 0 ≤ *n* < *N*.
Step 2.4 After all of the rows are processed in this way, repeat the process (Step 2.1–2.3) on columns.
Step 3: Compute Φ_*m*,*n*_ using Equation (6).
Step 4: Compute the inverse FFT of Φ_*m*,*n*_ to obtain *ϕ̄*_*i*,*j*_.

## 3. The Proposed Architecture

**Figure 1.**
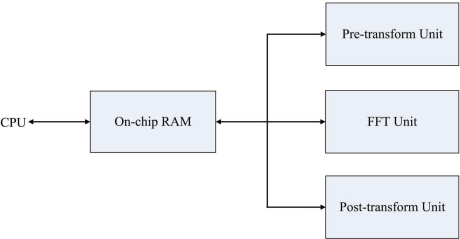
The proposed architecture for phase unwrapping.

Figure 1 shows the proposed architecture for the FFT-based phase unwrapping algorithm. As shown in the figure, the proposed architecture can be separated into four units: the pre-transform unit, the FFT unit, the post-transform unit, and the on-chip memory. Given *ζ*_*i*,*j*_, 0 ≤ *i* ≤ *N*, 0 ≤ *j* ≤ *N*, the goal of the pre-transform unit is to compute *γ*_*i*,*j*_. The FFT unit is then adopted for computing Γ_*m*,*n*_. After that, the post-transform unit is used for calculating Φ_*m*,*n*_. Finally, the FFT unit is used again for computing *ϕ̄*_*i*,*j*_ based on Φ_*m*,*n*_. The on-chip memory is used for storing both the original data and the intermediate and final results of the pre-transform unit, the FFT unit and the post-transform unit. Storing the original data and intermediate results in the on-chip memory effectively reduces the memory access time for the algorithm.

### 3.1. On-Chip Memory

The on-chip memory consists of two identical RAM modules. Each RAM module is able to store an (N+1) × (N+1) array. The RAM modules are shared by all the units in the proposed architecture. They are used to store the original or intermediate results produced by each unit. These results will then be used as the source data for subsequent operations. The employment of the on-chip memory is able to significantly reduce the memory access time for phase unwrapping.

### 3.2. Pre-Transform Unit

**Figure 2.**
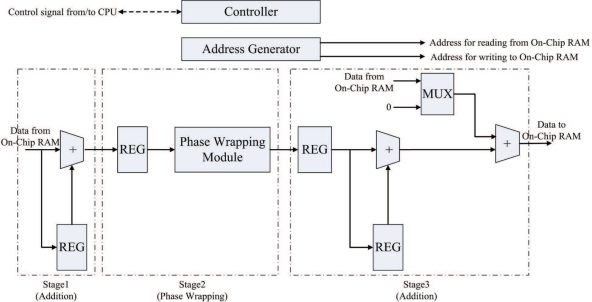
The architecture of pre-transform unit, where REG and MUX are the abbreviations of register and multiplexer, respectively.

The goal of the pre-transform unit is to implement Step 1 of the algorithm in hardware. Figure 2 shows the architecture of the pre-transform unit, which consists of controller, address generator, registers, adders, phase wrapping unit, and multiplexer. The address generator is used to generate addresses for reading the source data from the on-chip memory, and writing the results to the on-chip memory. The registers are used for the implementation of pipeline for enhancing the throughput. As shown in Figure 2, there are three stages in the pipeline. The first and the third stages are adders. The second stage is the phase wrapping unit, which is employed to ensure that the values of $\Delta_{i,j}^{x}$ and $\Delta_{i,j}^{y}$ lie in the interval (−*π*, *π*].

**Figure 3.**
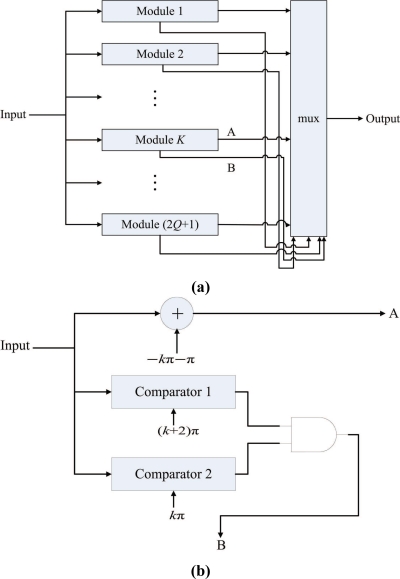
**(a)** The architecture of phase-wrapping unit in the pre-computation unit, **(b)** The architecture of each module *K* in the phase-wrapping unit.

Figure 3 depicts the architecture of the phase wrapping unit, which contains 2*Q* + 1 modules. Each module *i*, *i* = 1, ..., 2*Q* + 1, contains two comparators for determining whether a phase difference computed by Equation (2) lies in the interval (−(2*Q* + 1)*π* + 2(*i* − 1)*π*, −(2*Q* + 1)*π* + 2*iπ*]. The phase difference is first broadcasted to all the modules. After the interval in which the phase difference lies is identified, the phase wrapping operation is then performed accordingly so that the resulting wrapped phase difference lies in (−*π*, *π*].

**Figure 4.**
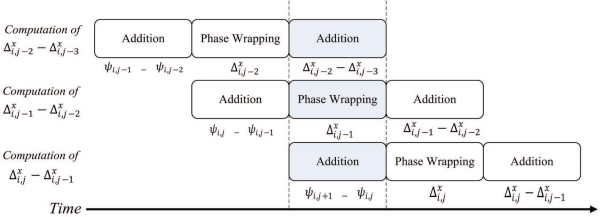
The timing diagram for pipeline operation at the first step of pre-transform unit.

**Figure 5.**
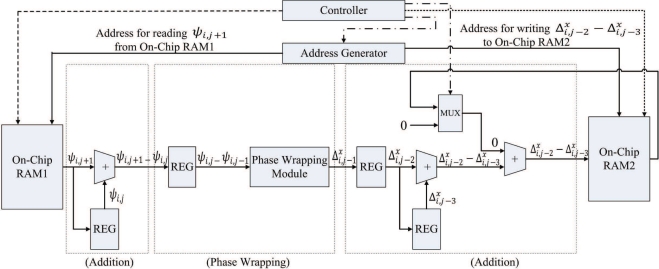
The input/output to each stage of the pipeline for the shaded time interval marked in the Figure 4.

The source data for the pre-transform operations, *ζ*_*i*,*j*_, 0 ≤ *i* ≤ *N*, 0 ≤ *j* ≤ *N*, are stored in the on-chip RAM 1. The pre-transform unit then produces *γ*_*i*,*j*_, 0 ≤ *i* ≤ *N*, 0 ≤ *j* ≤ *N*, and stores them in both the on-chip RAM 1 and RAM 2. The pre-transform unit computes *γ*_*i*,*j*_ in two steps. At the first step, *ζ*_*i*,*j*_ is retrieved from the on-chip RAM 1 to compute $\Delta_{i,j}^{x}-\Delta_{i-1,j}^{x}$, which will then be stored in the on-chip RAM 2. Figure 4 shows the timing diagram for the pipeline operation of the pre-transform unit at the first step. Figure 5 reveals the input/output to each stage of the pipeline for the shaded time interval marked in the Figure 4.

**Figure 6.**
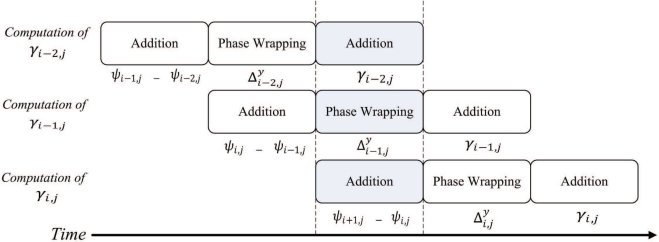
The timing diagram for pipeline operation at the second step of pre-transform unit.

**Figure 7.**
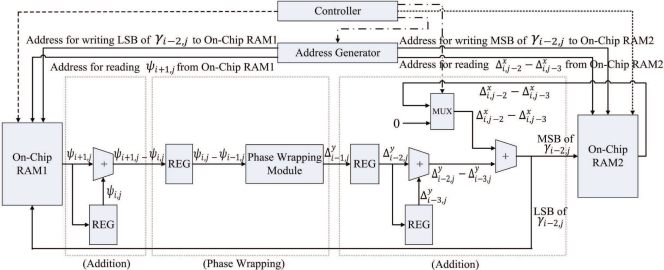
The input/output to each stage of the pipeline for the shaded time interval marked in the Figure 6.

At the second step, *ζ*_*i*,*j*_ is retrieved again from the on-chip RAM 1 to compute $\Delta_{i,j}^{y}-\Delta_{i,j-1}^{y}$. In addition, $\Delta_{i,j}^{x}-\Delta_{i-1,j}^{x}$ is retrieved from the on-chip RAM 2. The summation of ( $\Delta_{i,j}^{y}-\Delta_{i,j-1}^{y}$) and ( $\Delta_{i,j}^{x}-\Delta_{i-1,j}^{x}$) forms *γ*_*i*,*j*_, which will then be stored back to the on-chip RAM 1 and RAM 2 for the subsequent FFT operations. Figure 6 shows the timing diagram for the pipeline operation of the pre-transform unit at the second step. The input/output to each stage of the pipeline for the shaded time interval marked in the Figure 6 is then depicted in Figure 7. Note that, at the final stage of the pipeline, the MSBs (most significant bits) and LSBs (least significant bits) of *γ*_*i*,*j*_ are stored in on-chip RAM 1 and RAM 2, respectively. By storing the results to two modules, the computation precision is then doubled for subsequent operations.

Note that, as shown in Figures 5 and 7, the input to the pre-transform circuit is *ψ*_*i*,*j*_, which is the mirror reflected version of *ζ*_*i*,*j*_ in accordance with Equation (1). From Equations (2)–(5), it follows that the computation of *γ*_*i*,*j*_ is based on *ψ*_*i*,*j*_ instead of *ζ*_*i*,*j*_. When 0 ≤ *i* ≤ *N*, 0 ≤ *j* ≤ *N*, because *ψ*_*i*,*j*_ = *ζ*_*i*,*j*_, the *ζ*_*i*,*j*_ stored in on-chip RAM 1 is used as *ψ*_*i*,*j*_. Otherwise, *ψ*_*i*,*j*_ should be computed using Equation (1). It can be easily shown that only *ψ*_−1,_*_j_*, *ψ_N_*_+1,_*_j_*, *ψ*_*i*,−1_, and *ψ*_*i*,*N*+1_, 0 ≤ *i* ≤ *N*, 0 ≤ *j* ≤ *N* actually require mirror reflection for the computation of *γ*_0,*j*_, *γ*_*N*,*j*_, *γ*_*i*,0_ and *γ*_*i*,*N*_. Using Equation (1), it follows that *ψ*_−1,*j*_ = *ζ*_1,*j*_, *ψ*_*N*+1,*j*_ = *ζ*_*N*−1,*j*_, *ψ*_*i*,−1_ = *ζ*_*i*,1_, and *ψ*_*i*,*N*+1_ = *ζ*_*i*,*N*−1_, it is not necessary to design a circuit for mirror reflection for the pre-transform unit. We only have to reconfigure the address generator in the unit so that when *ψ*_−1,*j*_, *ψ*_*N*+1,*j*_, *ψ*_*i*,−1_, or *ψ*_*i*,*N*+1_ are desired, the address of *ζ*_1,*j*_, *ζ*_*N*−1,*j*_, *ζ*_*i*,1_ or *ζ*_*i*,*N*−1_ will be delivered to on-chip RAM 1, respectively.

Another advantage of the employment of address generator is that it is able to generate multiple addresses for the concurrent read and write accesses of the on-chip memory. Multiple address generation is essential for the implementation of the pipeline in the pre-transform unit. For the shaded time interval indicated in Figure 7, the retrieval of *ψ*_*i*+1,*j*_ and $\Delta_{i,j-2}^{x}-\Delta_{i,j-3}^{x}$ are required at stages 1 and 3, respectively. In addition, the computation result at stage 3, *γ*_*i*−2,*j*_, should also be written to the RAM 1 and RAM 2. As shown in Figure 7, the address generator sends three different addresses to the on-chip memory for the concurrent access: address for reading *ψ*_*i*+1,*j*_ from RAM 1, address for reading $\Delta_{i,j-2}^{x}-\Delta_{i,j-3}^{x}$ from RAM 2, and address for writing *γ*_*i*−2,*j*_ to RAM 1 and RAM 2. Other alternatives for memory accesses are based on CPU or direct memory access (DMA). However, because there is only one memory access at a time, using the CPU or DMA-based memory accesses for the proposed pipeline architecture may be difficult.

### 3.3. FFT Unit

**Figure 8.**
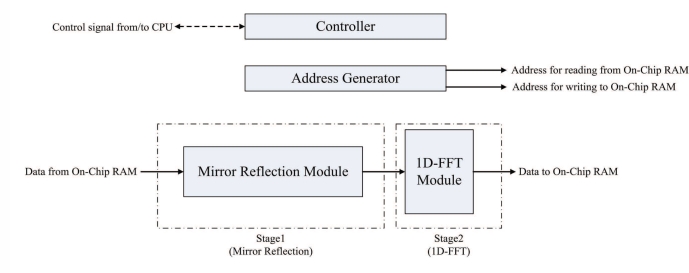
The architecture of FFT unit.

The FFT unit is employed for implementing Steps 2 and 4 of the algorithm. Figure 8 shows the architecture of the FFT unit, which contains controller, address generator, mirror reflection module and one-dimensional FFT (1D-FFT) module. The FFT unit reads the source data from both on-chip RAM 1 and RAM 2. The results produced by the FFT unit are then stored back to on-chip RAM 1 and RAM 2 to replace the source data.

In the FFT unit, each row of *γ*_*i*,*j*_ is loaded from the on-chip memory one at a time. The FFT unit then writes the computational results directly back to the same row in the on-chip memory. After the row operations are completed, the column operations will proceed in the same manner. After the completion of all the column operations, the array stored in the on-chip RAM is Γ_*m*,*n*_, the two-dimensional FFT of *γ*_*i*,*j*_.

From Step 2.1 of the phase unwrapping algorithm, it follows that the mirror reflection is required before the 1D-FFT transform (or inverse transform). The mirror reflection module is a 2*N*-stage shift register. The input to the shift register is located at the stage *N* + 1. The first *N* + 1 stages of the shift register are able to operate in two directions for the implementation of mirror reflection. The output of the shift register is connected to the 1D-FFT module. The proposed module operates in two phases using the shift register. At the first phase, each data point entering the mirror reflection module is shifted in two directions. After all the data points in a row have entered the shift register, all the data points are shifted right one at a time to the output at the second phase.

We use Altera FFT MegaCore function [26] to implement the 1D-FFT module. The transform length of the FFT is 2*N*. The 1D-FFT module has single data input and single data output. The input/output dataflow of the module operates in streaming mode, allowing the continuous process of input data stream, as well as producing the continuous output data stream. In addition, the module contains only one butterfly processor. Therefore, it requires only a single complex multiplier for the FFT implementation [26]. The area cost can then be minimized.

**Figure 9.**
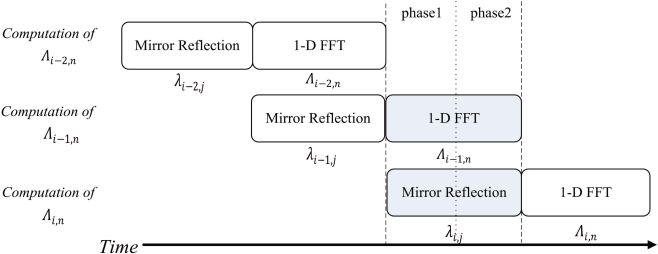
The timing diagram for pipeline operation of FFT unit.

**Figure 10.**
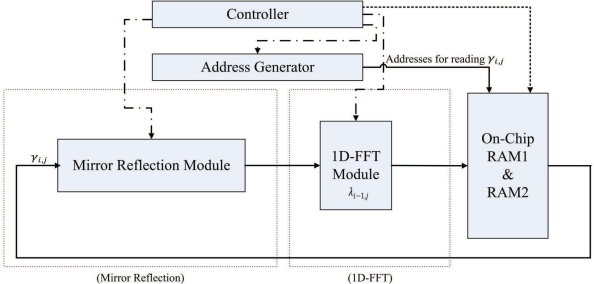
The operation of phase 1 at each stage of the pipeline for the shaded time interval marked in the Figure 9.

**Figure 11.**
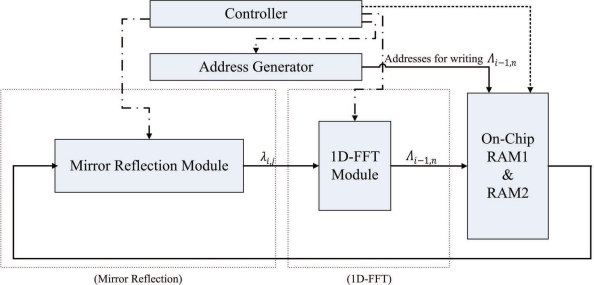
The operation of phase 2 at each stage of the pipeline for the shaded time interval marked in the Figure 9.

The FFT unit is able to operate as a two-stage pipeline, where the first stage is mirror reflection, and the second stage is 1D-FFT. Figure 9 shows the timing diagram for the pipeline operation of the FFT unit for the rows of the array *γ*_*i*,*j*_. Note that the operation of each stage of FFT unit is separated into two phases. Both the stages will operate at the same phase at the same time. Figures 10 and 11 show the operation of phase 1 and phase 2 at each stage, respectively. As shown in Figure 10, at the phase 1 of the mirror reflection, a row of *γ*_*i*,*j*_ (e.g., *i*-th row) is loaded from on-chip RAM for computing *λ*_*i*,*j*_, 0 ≤ *j* < 2*N*. At the same time, the phase 1 of 1D-FFT module uses *λ*_*i*−1,*j*_, 0 ≤ *j* < 2*N* as the input for computing Λ_*i*−1,*n*_, 0 ≤ *n* < 2*N*. The mirror reflection module then delivers *λ*_*i*,*j*_, 0 ≤ *j* < 2*N* to the 1D-FFT module at its phase 2 operation. At the same time, the 1D-FFT module sends the Λ_*i*−1,*n*_, 0 ≤ *n* < *N*, to the on-chip RAM, as depicted in Figure 11.

Note that the FFT unit is also used for the computation of inverse 2D-FFT of Φ_*m*,*n*_. The data stored in the on-chip memory is Φ_*m*,*n*_. The 1D-FFT module will operate as the 1D inverse FFT for the input data. The FFT unit will then produce *ϕ̄*_*i*,*j*_ to the on-chip memory.

### 3.4. Post-Transform Unit

**Figure 12.**
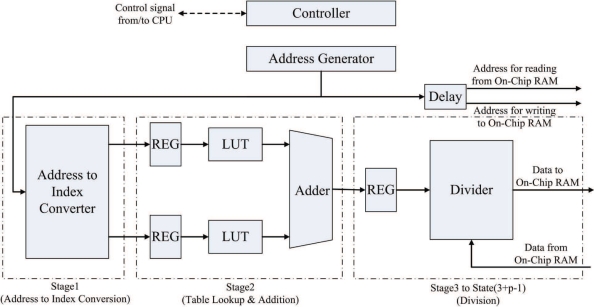
The architecture of post-transform unit.

The post-transform unit is used for the hardware computation of Step 3 in the algorithm. Therefore, the objective of the post-transform unit is to realize Equation (6) in hardware. Figure 12 shows the architecture of the post-transform unit, which consists of two cosine computation modules, a divider, and adders. The goal of the two cosine computation modules is to compute cos(*πm/N*), *m* = 0, ..., *N* − 1, and cos(*πn/N*), *n* = 0, ..., *N* − 1, respectively. Since *m* and *n* only takes *N* possible values for the cosine transform, the two modules can be implemented as a simple look-up table (LUT), consisting of *N* entries. The *i*-th entry of the table in the two cosine computation modules contains the value of cos(*πi/N*).

Although LUTs can be used for the implementation of cosine modules, they may be difficult to be used for the design of divider in the post-transform unit. From Equation (6), we can see that the nominator and denominator of the divider are all real numbers. Consequently, the number of entries of the LUT will be very high when it is used for divider design. A general divider which accepts real numbers as the inputs is then desired. The divider in the architecture is implemented by Altera Floating Point Megafunction ALTFP_DIV [27]. The divider is separated into *p* pipeline stages to enhance the throughput of the post-transform unit.

Given Γ_*m*,*n*_ in the on-chip memory, the post-transform unit operates as follows. The unit loads the FFT coefficients Γ_*m*,*n*_ from the on-chip memory one at a time based on the raster scan order. To reduce the amount of bus traffic, the address delivered to the on-chip memory for loading Γ_*m*,*n*_ is also delivered to the post-transform unit for extracting the indices *m* and *n*, which are then delivered to the cosine computation modules for finding cos(*πm/N*) and cos(*πn/N*). Both Γ_*m*,*n*_ loaded from the on-chip memory and (2 cos(*πm/N*) + 2 cos(*πn/N*) − 4) computed by the adders are then used as the input to the divider for computing Φ_*m*,*n*_. The output of the divider is then stored directly back to the on-chip memory.

**Figure 13.**
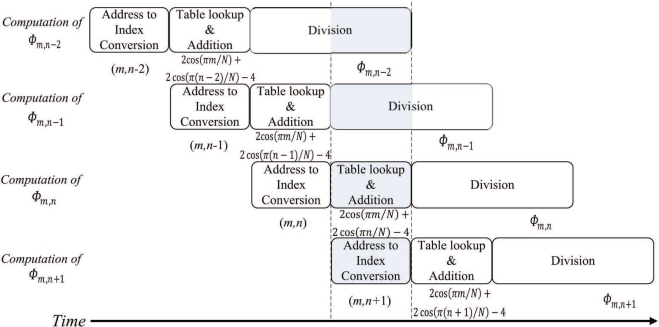
The timing diagram for pipeline operation at post-transform unit for *p* = 2.

**Figure 14.**
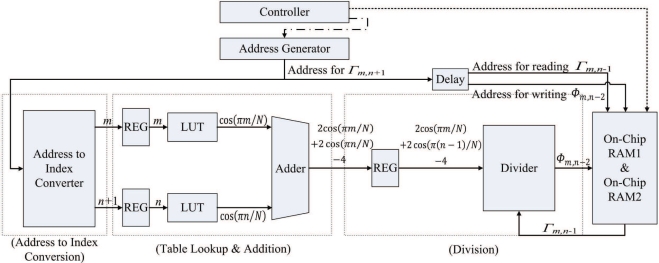
The input/output to each stage of the pipeline for the shaded time interval marked in the Figure 13.

**Figure 15.**
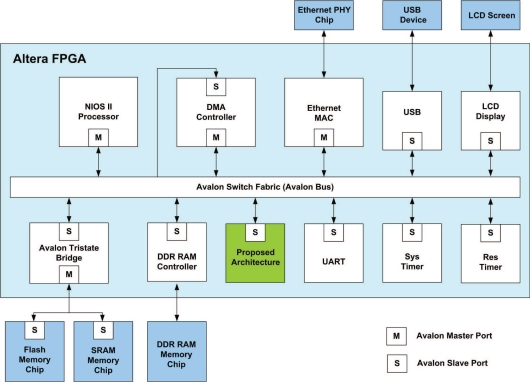
The SOPC system for phase unwrapping.

The post-transform unit is implemented as a (2 + *p*)-stage pipeline. As shown in Figure 12, the first stage performs the address to index conversion. That is, the address used for retrieving Γ_*m*,*n*_ from the on-chip RAM is used for computing indices *m* and *n* at this stage. The second stage of the pipeline computes cos(*πm/N*) and cos(*πn/N*) based on table look-up method. In addition, the second stage consists of an adder for computing (2 cos(*πm/N*) + 2 cos(*πn/N*) − 4). The third stage to the (2 + *p*)-th stage of the pipeline form a *p*-stage divider for computing Φ_*m*,*n*_. Figure 15 shows the timing diagram for the pipeline operation of the post-transform unit. The input/output to each stage of the pipeline for the shaded time interval marked in the Figure 13 is then depicted in Figure 14.

The major advantage of the design is that only the Γ_*m*,*n*_ is required from the input ports. The terms cos(*πm/N*) and cos(*πn/N*) can be obtained from the address for retrieving Γ_*m*,*n*_ from the on-chip RAM. Based on the address, the computation of cos(*πm/N*) and cos(*πn/N*) are then carried out by simple LUT. Consequently, the single address for retrieving Γ_*m*,*n*_ actually produces Γ_*m*,*n*_, cos(*πm/N*) and cos(*πn/N*) for computing Φ_*m*,*n*_ using Equation (6). This single-address-multiple-data scheme is beneficial for enhancing the computational speed of the post-transform unit.

### 3.5. Analysis of Area Costs and Speed

Two types of performance are considered in this paper: the latency and area complexities. The latency of each unit is defined as the time required for finishing the operations of that unit. Because the arithmetic operators and storage cells are the basic building blocks for the architecture, the area complexities are separated into 2 categories: the number of arithmetic operators, and the number of storage cells. The arithmetic operators consist of adders, multipliers, and dividers. The storage cells contain registers, ROM cells and RAM cells.

Table 1 summarizes the area complexities and latency of the proposed architecture. Recall that the images considered in the proposed architecture are of image resolution (N+1) × (N+1). It can then be observed from Table 1 that the number of arithmetic operators in the proposed architecture is independent of the image resolution. In addition, the number of storage cells grows linearly with the image resolution.

The number of arithmetic operators is independent of the image resolution because each unit in the proposed architecture only uses a fixed number of adders, multipliers and/or dividers, independent of *N*. For example, the FFT unit only employs one complex multiplier in the 1D FFT module. The post-transform unit also adopts only one divider.

The number of storage cells used by the proposed architecture increases with the image resolution. For the FFT unit, because the mirror reflection module contains 2*N* registers, its complexity is *O*(*N*). For the post-transform unit, the size of tables for cosine transform is also proportional to *N*. The number of storage cells in the on-chip RAM1 and RAM2 is (N+1) × (N+1). Its complexity therefore is *O*(*N*^2^), and grows linearly with the image resolution.

To evaluate the time complexity, we first note that the pre-transform and post-transform units need to perform additions and division to each of the (*N* + 1) × (*N* + 1) input data points, respectively. Since the number of adders and dividers are independent of *N*, the latency of these units is *O*(*N*^2^). For the 2D FFT and 2D IFFT operations, the latency is given by *O*(*N*^2^*logN*).

### 3.6. Software-Hardware Co-Design

The proposed architecture is used as a custom user logic in a SOPC system consisting of softcore NIOS CPU [28], and the proposed architecture, as depicted in Figure 15.

The objective of NIOS CPU is to control the data flow of the proposed architecture. Note that the on-chip RAM in the proposed architecture provides the source data for pre-transform unit, FFT unit and post-transform unit. The on-chip RAM also stores the computation results from these units. To ensure that the data in on-chip RAM is delivered to the correct unit, and the computation results of each unit can be sent to the on-chip RAM, the CPU is responsible for activating controller at each unit, and specifying the proper value in the status register in the on-chip RAM, which controls the multiplexer in the read and write ports of the memory.

As shown in Figure 15, the proposed architecture is connected to the Avalon bus for the communication with the NIOS CPU. A special interface circuit is designed for the connection. The interface circuit contains registers, address decoders and multiplexers so that the on-chip memory and the status registers in the controllers of all the units in the proposed architecture can be accessed by the NIOS CPU.

**Figure 16.**
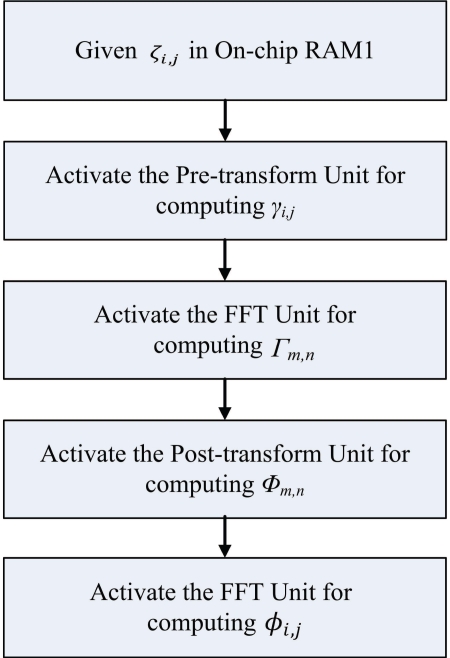
The flowchart of the software executing by the CPU.

Figure 16 shows the flowchart of the software executed by the CPU. It can be observed from Figure 16 that the software only involves the controller activation, as well as specifying the contents of status register in the on-chip RAM. Because of the simplicity of the software, the execution of the phase-unwrapping algorithm can be significantly enhanced.

## 4. Experimental Results

This section presents some experimental results of the proposed architecture. The design platform is Altera Quartus II with SOPC Builder and NIOS II IDE. The target FPGA device for the hardware design is Altera Stratix III EP3SL150.

The hardware resource utilization of each unit in the proposed architecture are revealed in Tables 2–4. The images resolutions considered in these tables are 129 × 129 (*i.e.*, N = 128), 257 × 257 (*i.e.*, N = 256) and 513 × 513 (*i.e.*, N = 512). There are three types of area costs considered in the experiment: adaptive logic modules (ALMs), embedded memory bits, digital signal processing (DSP) blocks. The ALMs are used for the implementations of arithmetic operators and storage cells. The embedded memory bits are used mainly for storage cells. The DSP blocks are used only for arithmetic operators such as dividers and multipliers. The target FPGA device Altera Stratix III EP3SL150 contains 56,800 ALMs, 5,630,976 embedded memory bits, and 384 DSP blocks.

It can be observed from Tables 2–4 that both the pre-transform and post-transform unit utilize only a small percentage of ALMs, embedded memory bits, and DSP blocks. The ALM utilization of post-transform unit grows with image resolution because the tables in the cosine computation modules are implemented by ALMs. The divider in the post-transform module is implemented by DSP blocks. The major hardware resource utilized by on-chip memory is the embedded memory bits, which grows linearly with *N*^2^. The FFT unit utilizes most of the ALMs, which are used for the design of mirror reflection module and 1D FFT module. The FFT unit uses DSP block only for the implementation of complex multipliers. Since there is only one complex multiplier in the 1D FFT module, independent of image resolutions. The DSP block utilization of FFT unit is therefore also independent of image resolutions.

Table 5 shows the total hardware resource utilization of the proposed architecture for images with different resolutions. It can be observed from the table that the increase in the ALM utilization is small when image resolution enlarges. In fact, as the image resolution increases 16 folds (*i.e.*, from 129 × 129 to 513 × 513), the ALM utilization is only increased approximately 2 folds (*i.e.*, from 9,580 to 2,1212). The embedded memory utilization grows linearly with the image resolution. The DSP block utilization is independent of the image resolution. In addition to the area complexities of the entire architecture, the table consists of the area complexities of the entire SOPC using the proposed architecture as a custom user logic.

**Figure 17.**
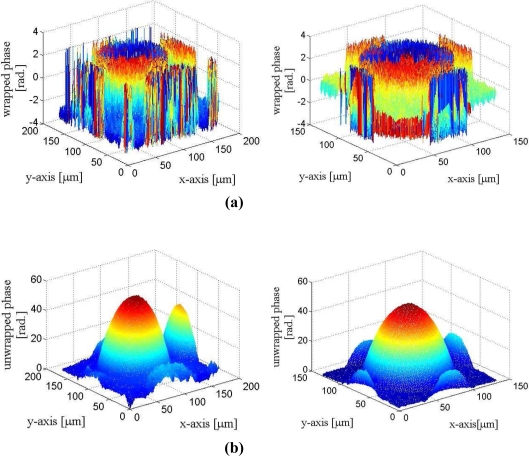
The phase unwrapping results for 257 × 257 images: **(a)** The phase wrapped image, **(b)** The phase unwrapped image produced by the proposed architecture.

The execution time of each step of phase unwrapping algorithm implemented by the proposed architecture for various image resolutions is shown in Table 6. The execution time is measured by the SOPC platform with 500 MHz NIOS softcore CPU. The proposed architecture is used as the accelerator, as shown in Figure 17. It can be observed from the table that the FFT and IFFT are the most time consuming operations. In particular, when image resolution is 513 × 513, both the FFT and IFFT consume 71.9 % of the total computational time. These results are consistent with those shown in Table 1, where the FFT unit has the highest time complexity.

Although the division and cosine operations are required in the post-transform unit, the computation time is low and comparable to that of the pre-transform unit. For a 513 × 513 image, the post-transform unit consumes only 15.7% of the total computation time. The fast computation is due to the efficient single-address-multiple-data operations as revealed in Figures 14 and 15. The single address for retrieving Γ_*m*,*n*_ actually produces Γ_*m*,*n*_, cos(*πm/N*) and cos(*πn/N*) for computing Φ_*m*,*n*_ in a pipeline fashion. As a result, the architecture is able to minimize number of memory accesses while maintaining high throughput.

Table 7 shows the speed of the proposed phase unwrapping architecture for images with various resolutions. The speed of its Matlab software counterpart running on the 2.8 GHz Intel I7 quad-core processor with 4 GB DDR III is also included in the table for comparison purpose. We can see from Table 7 that the proposed hardware circuit operates at significantly faster speed. The speedup over its software counterpart grows with the image resolutions. As the image resolution reaches 513 × 513, the speedup becomes 605. Note that the total computation time of phase unwrapping for 513 × 513 images is only 8.9 ms. This implies that the proposed architecture can support rendering with frame rate 100 fps. The proposed architecture therefore can be effectively employed for embedded DHM requiring rendering with high frame rate.

Table 8 lists the computation time of various existing phase unwrapping implementations. The direction comparisons of these implementations may be difficult because these implementations are based on different phase unwrapping algorithms with different image resolutions. In addition, these implementations are realized by different platforms. Nevertheless, we can still see from Table 8 that the proposed is an effective alternative for phase unwrapping when the computation time is an important concern.

**Figure 18.**
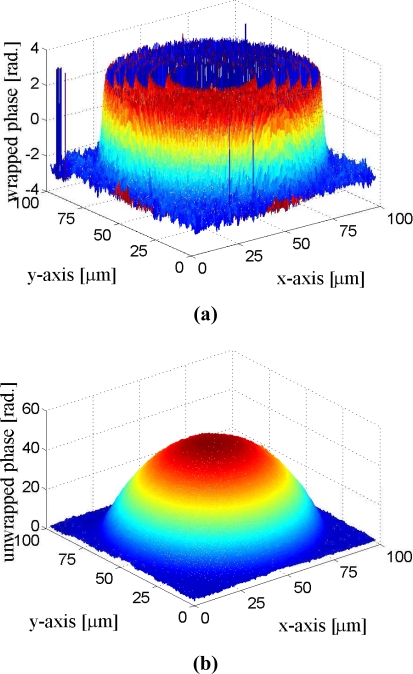
The phase unwrapping results for 513 × 513 image: **(a)** The phase wrapped image, **(b)** The phase unwrapped image produced by the proposed architecture.

Figures 17 and 18 show the phase unwrapping results of the proposed architecture. The images considered in the experiments are produced by the DHMs in the IOP lab at the Institute of Electro-Optical Science and Technology, National Taiwan Normal University. In the experiments, microlens made by SUSSA (with radius of curvature 120 microns) are tested. The image resolution is 257 × 257 and 513 × 513 for the images shown in Figures 17 and 18, respectively. To evaluate the accuracy of the reconstruction, the radius of the curvature in the phase unwrapped results are measured, and are compared with the actual radius of the curvature of the microlens. The measured radius of curvature of the two microlens in Figure 17 are 121.0 and 121.1 microns, respectively. The measured radius of curvature of the microlen in Figure 18 is 119.7 microns. The maximum error of the unwrapped results therefore is only 1.1 microns for the measurement of radius of curvature.

## 5. Concluding Remarks

The proposed architecture has been found to be effective for phase unwrapping. It utilizes low hardware resources. Only a single divider and complex multiplier is used in the architecture. The utilization of DSP blocks therefore is minimized. The ALM and memory bits utilization also only grow linearly with the image resolutions. Each unit in the architecture is implemented in a pipeline fashion for enhancing the throughput. The architecture therefore has fast computation speed. In particular, when the image resolution is 513 × 513, the computation time is only 8.1 ms. The speedup attains 605 over its software counterpart. The architecture is able to support frame rate above 100 fps for embedded DHM rendering. The architecture is an effective alternative for the implementation of embedded DHM systems where low hardware resource utilization, high image resolution and high image rendering rate are desired.

## Figures and Tables

**Table 1.** Area complexities and latency of the proposed architecture with respect to the image resolution (N+1) × (N+1), where the function *O*, termed big *O* function, is used to indicate the asymptotic complexities of the architecture.

	Pre-Transform	FFT	Post-Transform	On-Chip Memory	Overall
Arithmetic Operators	*O*(1)	*O*(1)	*O*(1)	0	*O*(1)
Storage Cells	*O*(1)	*O*(*N*)	*O*(*N*)	*O*(*N*^2^)	*O*(*N*^2^)
Latency	*O*(*N*^2^)	*O*(*N*^2^ log *N*)	*O*(*N*^2^)		*O*(*N*^2^ log *N*)

**Table 2.** The ALM utilization of each unit in the proposed architecture for various image resolutions.

Image Resolutions	Pre-Transform	FFT	Post-Transform	On-Chip Memory
129 × 129	197	8,358	889	136
257 × 257	201	10,532	959	178
513 × 513	221	19,049	1,641	301

**Table 3.** The embedded memory bit utilization of each unit in the proposed architecture for various image resolutions.

Image Resolutions	Pre-Transform	FFT	Post-Transform	On-Chip Memory
129 × 129	0	61,440	4,608	299,538
257 × 257	0	122,880	4,608	1,188,882
513 × 513	0	233,472	4,608	4,737,042

**Table 4.** The DSP block utilization of each unit in the proposed architecture for various image resolutions.

Image Resolutions	Pre-Transform	FFT	Post-Transform	On-Chip Memory
129 × 129	0	24	16	0
257 × 257	0	24	16	0
513 × 513	0	24	16	0

**Table 5.** The total area costs of the proposed architecture for various image resolutions.

	Proposed Arch.			Entire SOPC		

Image Resolutions	ALMs	Embedded Memory Bits	DSP Blocks	ALMs	Embedded Memory Bits	DSP Blocks
129 × 129	9,580/56,800 (17%)	365,586/5,630,976 (6%)	40/384 (10%)	17,081/56,800 (30%)	968,722/5,630,976 (17%)	44/384 (11%)
257 × 257	11,870/56,800 (21%)	1,316,370/5,630,976 (23%)	40/384 (10%)	20,905/56,800 (36%)	1,916,434/5,630,976 (34%)	44/384 (11%)
513 × 513	21,212/56,800 (37%)	4,975,122/5,630,976 (88%)	40/384 (10%)	28,568/56,800 (50%)	5,085,778/5,630,976 (90%)	44/384 (11%)

**Table 6.** The execution time of the proposed phase unwrapping architecture for various image resolutions.

Image Resolutions	Pre-Transform	FFT	Post-Transform	Inverse FFT	Total
129 × 129	0.1 (ms)	0.3 (ms)	0.2 (ms)	0.3 (ms)	0.9 (ms)
257 × 257	0.3 (ms)	0.9 (ms)	0.4 (ms)	0.9 (ms)	2.5 (ms)
513 × 513	1.1 (ms)	3.2 (ms)	1.4 (ms)	3.2 (ms)	8.9 (ms)

**Table 7.** The execution time of the proposed phase unwrapping architecture for various image resolutions.

Image Resolutions	Proposed Architecture	Software Counterpart	Speedup
129 × 129	0.9 (ms)	468 (ms)	585
257 × 257	2.5 (ms)	1504 (ms)	601
513 × 513	8.9 (ms)	5389 (ms)	605

**Table 8.** The execution time of different phase unwrapping implementations.

Implementations	Computation Time	Image Resolutions	Platforms
Proposed Architecture	8.9 (ms)	513× 513	FPGA (Altera Stratix III EP3SL150)
[19]	672 (ms)	512× 512	GPU (NVIDIA Geforce 8800GTX)
[21]	2.8 (s)	640× 480	GPU (NVIDIA Geforce 8800GTX)
[20]	24.7 (s)	1, 024× 512	FPGA (Xilinx Vertex II Pro)
